# Feedback with feelings: the human complexity of expressing judgements about performance

**DOI:** 10.1007/s10459-025-10477-w

**Published:** 2025-09-25

**Authors:** Margaret Bearman, Joanne Hilder, Damian Castanelli, Elizabeth Molloy, Chris Watling, Robyn Woodward-Kron, Rola Ajjawi

**Affiliations:** 1 Deakin University, Melbourne, Australia; 2 Southern Cross University, Gold Coast, Australia; 3 Monash University, Melbourne, Australia; 4 University of Melbourne, Melbourne, Australia; 5 Royal College of Surgeons and Physicians of Canada/ Western University, London, Canada; 6 University of British Columbia, Vancouver, Canada

**Keywords:** Feedback, Emotions, Postgraduate training, Post-qualitative research

## Abstract

Feedback is an emotional business; evoking optimism, fear or disappointment, which in turn can lead to engagement, further feedback seeking or avoidance. Emotions therefore are not just background noise but are fundamental to the experience. Through conceptualising emotions as embodied, social and complex, we seek to better understand how feelings work within feedback in clinical education, beyond an individual ‘managing’ their emotions. In this post-qualitative study, we ask: How does the interplay between feelings and feedback unfold in specialty medical training? To this end, we conducted a focussed ethnography of feedback in intensive care medicine and surgical training in Australian tertiary-care hospitals. Thinking with theory, we traced how trainees’ feelings move within and between feedback encounters through observation-based field notes and interview transcripts. We provide thick description of how feedback is saturated with feeling, integrating our findings with discussion. Supervisors expressed their judgements about trainee performance *as* feelings, *through* feelings and *about* feelings. And trainees responded with feelings of their own. Formal feedback particularly intensified feelings, which ‘stuck with’ trainees, leading to action, including avoidance. Feelings served to strengthen relationships and reinforce social hierarchies both within and beyond the supervisor-trainee dyad. We infer that the judgements made in and around feedback – such as appraisal of source credibility, assessment of performance quality, and deciding future actions – are themselves fundamentally entangled with feelings. A first step to remedy the desire to ‘manage’ emotions through ‘putting them away’ is to acknowledge their presence in clinical learning environments.

## Introduction

Feedback can spark joy. It also leads to disappointment or indifference or mistrust; feedback can be associated with shame, despair and deep fury (Bearman et al., 2024; Bynum et al., 2019; Gingerich et al., 2022). Over time, such feelings may prompt behaviours such as feedback seeking and/or avoidance. The medical education literature most often represents emotion in feedback as an internal psychological state, which can be “managed” (Ajjawi et al., 2022, p. 480). The tacit corollary is that learners and educators can overcome negativity; with enough willpower, rational brains will conquer irrational hearts. We contend that emotions are more than an educational sidenote. They are not disconnected and manageable cognitive states, they are *felt* in the body rather than known in the head (von Scheve, 2018). By studying the interplay of feelings with conversations about performance in clinical environments, we can start to understand (and do) feedback differently.

Many feedback studies highlight the critical role of emotions (what might also loosely be called feelings or affect) in medical education (Ajjawi et al., 2022). As both feedback and emotions are contested terms (Lipnevich & Panadero, 2021; Olson et al., 2020), we will define both. We first establish what we mean by *feedback*. In clinical education feedback is increasingly seen as relational, rather than a matter of information provision. Studies highlight the significance of trainee-supervisor relationships (Bing-You et al., 2018; Ramani et al., 2020; Telio et al., 2015) and the role of colleagues, patients and clinical cues (Bearman et al., 2022; Van Der Leeuw et al., 2018; Watling et al., 2012). For us, the central feature of feedback is that it encompasses the interactions accompanying a judgement about the trainee performance. Thus, feedback can take place in a formal appraisal, in a corridor, or during a hands-on patient care task such as an operative procedure. While the supervisor is the most common source of these judgements about performance – and surrounding conversations – feedback can take place with peers, other medical staff, other health professionals and patients (Bearman et al., 2023; Coelho et al., 2022).

A small subset of the literature explicitly explores emotions in feedback as a primary focus, and in these, emotion is generally taken as a psychological state that influences learning. For example, Eva and colleagues (2012) report on how the “fear of looking really stupid” (p.19) and avoidance of “negative emotions” (p. 23) might lead learners to avoid or discount certain feedback messages. In a similar vein, Sargeant and colleagues (2008) report how negative emotions can interfere with learning from feedback messages. However, a 2018 study of emotional talk in feedback narratives (Dennis et al., 2018, p. 75) recognises the emotionality in *both* supervisor and trainee accounts, with a call to “better understand how emotion plays a role in feedback as a complex social process”.

To understand emotions within feedback as a complex social process, we must closely interrogate what we mean by *emotions*. As mentioned, the feedback literature mostly represents emotions as an internal state – happy or sad or angry – thus learners can “manage their emotional equilibrium” to maximise their learning (Carless & Boud, 2018). However, as mentioned before, people often *feel* in the body before they know in the head (Massumi, 2021; von Scheve, 2018). For example, if something scares us, we recoil, the hairs stand up on the back of our neck, and our hearts race. These responses change how we think, speak and act (Brown & Stenner, 2001) and this in turn, changes how other people think, speak and act (Fox, 2015). Someone who observes our fear, may step in to comfort us, and in doing so, their own hearts may start racing, taking our fear into their own bodies. This is far more complex than being in a state such as happiness or sadness. To acknowledge this conceptual leap – from the cognitive to the embodied and social – we employ the word *feelings*.

We use *feelings* to capture entangled and multiple conceptualisations associated with emotionality and affective experiences. Massumi (2021) delineates the conscious experience of a feeling (e.g. I’m feeling happy); from the embodied state of a feeling before it is even articulated or experienced (e.g. my unconscious grin); and from how these feelings affect others (e.g. your unconscious smile in response to my grin and your conscious recognition of my apparent happiness). Therefore for us, feelings are *embodied* - matters of sensation, body language, speech and action – and *relational* – produced in relation to something or someone (Mehrabi, 2020) – and *social* (von Scheve, 2018). By the latter, we mean that feelings take place within socially and culturally prescribed relations (e.g. teacher-student or nurse-doctor), with different sanctioned activities and identities (von Scheve, 2018). Consider how differently people respond to a baby or a forty year old man crying in a supermarket. A social position grants a particular type of person permission to have particular feelings.

How can we understand the role of feelings in feedback in clinical contexts? In medical education, feedback is enmeshed within the social structures of the clinical context (Bearman et al., 2023; Cantillon et al., 2022). Thus, feedback conversations take place in a social and material web of interacting social structures, objects and spaces (Bearman et al., 2023). Certain social positions permit certain feelings in clinical environments. For example, feelings of loss or grief are associated with being a patient’s family member visiting the intensive care unit (ICU) but are less legitimised for the ICU registrar. However, this does not mean that the registrar does not feel in response to the patient’s condition or to the family member’s distress. Feelings “stick” and “intensify” and “circulate” (Ahmed, 2013). That is to say, feelings can persist, they can become stronger, and they can move from one person to another. Feelings also “affect” relations, bringing actors together or pushing them apart (Ahmed, 2013). These theoretical concepts can be illustrated by a clinical feedback encounter where the supervisor speaks harshly to a trainee. Feelings of shame or anger or guilt associated with the feedback can persist, grow stronger, and be *felt* by the trainee’s colleagues. As a consequence, the trainee starts avoiding any situation where they must work with the supervisor, and the colleagues who observed the encounter begin to watch the trainee closely to see if they make any errors.

Using this type of theoretical framing – embodied, social and complex – may help better understand how feelings work within feedback. Everyone intuitively knows feelings are significant. Beyond telling trainees and educators to manage their emotions, what more can we do?

This study seeks to provide insights into the interplay between feelings and feedback. Our focussed ethnography of feedback is set in intensive care medicine and surgical training in large Australian tertiary-care hospitals; in it we trace how trainees’ feelings move within and between feedback encounters. We ask the research question: How does the interplay between feelings and feedback unfold in specialty medical training?

## Methods

### Methodology

In this study we trace feelings in action within feedback, framed by sociomaterial theories of emotion and affect as outlined above, and interrogating a corpus of data that includes observation, text and audio. We gathered this corpus through a focussed ethnography (Knoblauch, 2005), whereby we sought to observe and explore feedback in specialty training environments. This type of ethnography narrows its exploration so that field work is focussed on the phenomenon of interest, and investigators are at least somewhat acquainted with the context and culture being studied (Andreassen et al., 2020; Knoblauch, 2005).

We position this work in the post-qualitative, new materialist movement which builds on the key works of St Pierre and Lather (Lather & St. Pierre, 2013; St Pierre, 2014; St. Pierre, 2019). Post-qualitative research moves away from prescriptive procedures and methodological rigidity, towards more creative theoretical approaches that disrupt practice and guide new ways of being (St Pierre, 2014). To this end we are employing methods that embrace subjectivity, reject linearity of research design and process, and emphasise lived experience (Cheshire, 2016). Post-qualitative research is driven by theory. We ‘think with theory’ (Jackson & Mazzei, 2011) to inform our analysis. Data that are dense and multi-layered can be ‘plugged in’ to different theories, thus opening up knowledge rather than ‘applying’ theory that might foreclose or simplify (Jackson & Mazzei, 2011).

### Data generation

We sought to observe feedback encounters between trainees and supervisors in two specialties, surgery and intensive care medicine, in two Australian hospitals. We supplemented these observations with contemporaneous field notes and diagrams and with short trainee interviews audio-recorded either immediately after or separately as a debriefing. On occasion, supervisory staff were interviewed. The observer also made audio diaries or on occasion recorded debriefings with participants’ colleagues. The observations and interviews took place in an Australian health service during the pandemic, which imposed some minor restrictions on the study in terms of access to environments. We observed four intensive care trainees and three surgical trainees and associated supervisors (both those who were in formal educational roles and those who were supervising ‘on the job’). For anonymity reasons, we do not list participant gender nor describe how many supervisors or trainees were involved but note there were broadly more women participants than men. We adjusted field notes to remove identifying contextual information including hospital and, on occasion, specialty. See Table 1 for a summary.

**Table 1:** Summary of data collected from participants

Data collected	ICU	Surgery	Total
Episodes of observation	15	11	26
Hours of observation	46.3	26.2	72.5
Interviews (trainee and supervisor)	16	8	24

We received ethical approval but for reasons of anonymity we do not list the relevant committee but note that it was certified with the National Health and Medical Research Council (NHMRC) of Australia thus guidelines are based on the Helsinki Declaration. While all participants consented to be observed and interviewed, the observer left immediately if they felt it was inappropriate to stay –for example in a stressful clinical situation. Participants were offered $100 AUD for their time and inconvenience. Non-participants who fell into the field of observation verbally consented to be included.

### Holistic, theoretically-informed analysis

Given the sociomaterial frame, we conceptualised feedback as located within a network of social relations. We focus here on *feedback encounters* (Esterhazy, 2018; Jensen et al., 2023), which we define as interactions surrounding a judgement about trainee performance. These interactions can be: conversational or behavioural; a once-off event or in a series; and one-to-one or in a group. Those making the judgement can include supervisors, peers and patients. We operationalise the encounter as including some form of conversation, but the judgement itself can be embodied and implicit rather than declarative. For example, suggesting to a trainee that they are now capable of working independently was classified as a type of feedback encounter: it is an interaction about a judgement on the trainee’s performance.

This primary analysis of our ethnographic data considered all sources in their totality and identified 20 feedback encounters. For each one, we collated descriptions from the sources that existed - as commonly there was both field note and interview data pertaining to the same event. We also included in our initial analysis another three situations that we considered ‘missed opportunities’ for feedback. We labelled these E1 to E23 and included other contextual data as relevant. To promote anonymity, we used the code E0, if we thought a piece of information might be identifying.

We analysed the 20 feedback encounters and 3 missed opportunities holistically following Ahmed (2013) by asking: what do feelings *do*? We considered how feelings *circulate* from one person to another by resonating (parallelling the feelings of the other), sticking (persisting) or intensifying (becoming more felt, but not necessarily the same). For example, one person’s expression of anger can intensify others’ feelings of sadness. We also noted how feelings pushed people together (enhanced relationality) or pulled them apart (diminished relationality). We started with two feedback encounters from our dataset and discussed them as a group. Then team members wrote short analyses for each encounter: (1) a narrative summary of what was happening drawing from the various data sources, and (2) inductive interpretive notes. The observation data often indicated how feelings were embodied; and we relied here on the observer interpreting and noting body language or their sense of how the participant was feeling. In interviews, participants often directly indicated how they were feeling and how these feelings were influencing their thoughts and actions. Team members discussed in pairs how the feelings and the network of relations were playing out in each of the encounters. Finally, the lead author collated all these analyses and re-interrogated them with the following questions:

- How do feelings circulate – intensify, resonate and stick?
- How do feelings push together or pull apart social relations?

### Representation of findings

Given the detailed theoretical and analytical frames, we do not separate findings from the discussion, aligning with the social studies of healthcare genre (Hoeyer & Bearman, 2023), where analysis, theory and literature are interwoven. To help the reader understand our insights, we describe our backgrounds as a mix of clinical and non-clinical professions, including anaesthesia, neurology, education, social work, and physiotherapy; all of us have doctorates in health professional education or similar. We include our reflexive responses within the discussion as fundamentally entangled in this knowledge generation. Strong feelings pervaded the feedback encounters and their intensity seeped from the original observations into our own bodies as we analysed the textual material. We noticed how we were affected through working with the data and how this affected our interpretations of it.

## Feedback encounters: tacit information, orchestration and judgement

Before we explore feedback and feelings in depth, we describe the types and shape of the feedback within our data. This study was designed as a complement to a separate, earlier, national interview study examining feedback cultures in specialty training (Bearman et al., 2023). The previous study detailed how feedback in Australian surgical and intensive care medicine education could be challenging, with trainees gaining only patchy information about their performance. The present dataset resonates with these findings. The most common kind of feedback experience was associated with particular patient care tasks, where judgements about performance could be entirely tacit such as “taking over”; these were more common in surgery than in intensive care medicine (Bearman et al., 2023). While collaborative planning for future feedback conversations is considered core to medical education feedback models (Weallans et al., 2021), planning was present but limited. For example, in our analysis of 20 feedback encounters, there were two involving plans – one encounter where the surgeon and trainee fulfilled a plan for the trainee to conduct gallbladder surgery with direct guidance (E13) and another encounter where a trainee moved to a new environment with the label of underperformance (E18). They report discussing with their new supervisor:

Hey, this is what I’ve been given as feedback. Can you help me work on it?… He now knows in the background what my feedback was and he’s then obviously watched and observed and made an assessment of why I potentially got that feedback. Now he’s going to work on my insecurities.

Feedback seeking behaviour was very striking in Bearman et al. (2023) where trainees often orchestrated opportunities for feedback and guidance on their performance – sometimes looking beyond their supervisor to other colleagues. Similarly in this study, we observed an intensive care medicine trainee asking a physiotherapist if she can “bag the patient”. The fieldnotes from E16 describe:

[the trainee] says that this would be good for her learning because in an emergency she needs to bag the patient but she has never done it before. The physio does a short teaching session […and] the nurse points to the screen to show [the trainee] where to see how [the trainee] and the patient [are] going. The physio says: *can you feel the bag sucking in when he takes a breath?* – *squeeze it evenly in - yep*,* that’s it.*

However in contrast to this type of direct guidance –and aligning with Bearman et al. (2023) – the trainees within this study received limited information about their overall progress in a form that made sense to them. In E2, a trainee reflects on critique within a generally successful end of term assessment:

I guess my communication has come up before …that’s probably my weakest area that needs to be worked on… more in the higher stress situations [how] to do that without coming across as more aggressive… I don’t think [I] was that bad this term. I think I’ve actually been quite good and improved comparatively to how I’ve been. … (E2).

After this mismatch between their supervisor’s judgement and their own, the trainee continues:

[In the conversation there wasn’t] really a method of how to do that with feedback or any specific examples given. It was the same feedback I got at the midterm… I’m not sure what I’ve done to show that [issue] again.” (E2).

This feedback encounter illustrates how feedback and judgement are interwoven. In this instance, the expressions of judgements about performance did not only come *with* feelings, but they were also *about* feelings. This whole section also illustrates how feedback information is both implicit and explicit. We structure the subsequent parts of this paper around the implicit and explicit expressions of judgements about performance within the feedback encounters.

## Judgement as feeling

Feedback encounters were saturated with feeling. Judgement itself appeared to be always a matter of feelings: supervisors expressed judgements *as* feelings and *through* feelings. And trainees responded with feelings of their own (i.e., they *circulated*).

Judgements about performance and associated circulating feelings were frequently observed in the feedback encounters embedded in day-to-day supervision of clinical practice. Our data illustrate how judgement and affect work together as *embodied*, *relational* and *social* interactions. For example, supervisor expressions of reassurance or encouragement, often so implicit that they might not be badged as feedback, *stuck* and *resonated* with the trainee. We interpret this occurred in the planned gallbladder surgery (E13). As the field notes describe:

[The trainee] mentioned that she has a *feeling* that the surgery went well. She could not really explain what informed that feeling but I suspect it was an intuitive judgement that was informed by the success of the operation and that the consultant was relaxed…

These types of interactions appeared to prefigure an increasing closeness between trainee and supervisor. After a consultant was observed agreeing with a trainee’s plan (and allowing them to practice unsupervised), the trainee reflects: “I feel that I’ve gained [the supervisor’s] trust with my learning or what he’s [the supervisor has] seen of my abilities sort of thing. It is reassuring.” (E11). This illustrates how the supervisor’s positive regard circulated to the trainee, with the trainee attuning their feelings towards those of their supervisor. This resonance suggests a likely closer relationship between supervisor and trainee, as also indicated by the word “trust”. For example, a trainee relates (E21):

There was one incident two months ago that the patient had a bad outcome as a result of a procedure that I did but it was one of those outcomes where it could have happened to anyone. I wasn’t aware immediately… the next morning one of the consultants took me aside in the office and told me that had happened but in a very neutral way and *non-judgemental but also very reassuring* and said, “No, this happens. This happened to people before. It happened to us.” [Italics added for emphasis]

The trainee goes on to describe how they responded, with “guilt obviously and obviously the fact that it happened to the patient…” But the reassurance of the “non-judgemental” tone – which we interpret is itself a judgement – changes how the feedback sticks: “now, it’s just a lesson to learn, to be aware of, to be vigilant.”

Other momentary expressions of judgement – of frustration or irritation or disappointment - also circulated within day-to-day feedback interactions. The impact of these expressions was not always immediately apparent but they could stick with the trainee for some time after. For example, in E1, the field notes states:

While the trainee is on the phone talking the supervisor is listening in. He states “just tell them our plan” in a loud voice and sounded slightly irritated. …When the participant hangs up the [supervisor] suggests “What was the goal of your calling them? For me the goal was an FYI [for your information] … Before the phone call you need to have a plan…I think you finished the call very well…” [The supervisor] then provides an example of ED [Emergency Department] calling to make a referral. He does acknowledge that it is a little easier for him because he is a consultant. The trainee replies: “Thank you – that’s a good point.

Later in the interview, while acknowledging the supervisor’s comments about how to improve communication, the trainee says:

There’s always an inner critic that says, “Guess what? Their perception of you has changed, they’re going to think that you’re not good at communicating.” … Have I tainted my whole- picture of how I should be and now it’s just seen as a weakness?

We interpret the sense of irritation and verbal comments as inseparable, and together they intensified the trainee’s own feelings about the judgement. Another trainee’s account:

[I was taking a] little bit of time to answer the question and I hear a sigh … which to me is inadvertently reinforcing that he’s disappointed in me and that I should know how to answer that question then and there and straight away. … (E0)

These things revealed in interviews were often hidden from the supervisor. For example, in E5 the trainee comments after the observation:

[I’m] left alone wondering, they were clearly unimpressed with that and why? What could I have done differently? What weren’t they happy with? I think, sometimes people forget that their body language tells a thousand words … (E5)

But the fieldnotes state:

…the consultant returns back to the ICU. He asks jokingly to the trainee and the resident “have you fixed them yet?” (This is a joke because all three patients have been in ICU for weeks.) Everyone seems to laugh but I’m not sure if it’s because they think what the consultant said is funny… (E5)

We interpret that this masking of anxiety (and dutiful laughter) simultaneously reinforces the supervisor-trainee hierarchical positions (Castanelli et al., 2022) but also pushes the supervisor and trainee further apart, preventing further feedback conversations. Moreover, the trainees may be intensifying their underlying feelings due to the effort of keeping the feelings contained.

To add further complication, supervisors could view their feelings as entirely benign, and their judgements as being in the trainees’ best interests. For example, a supervisor whose comments had upset a trainee noted:

I’m not convinced that [this specialty is for that trainee]. … My question has to be, why? Is it that they can’t [do a particular facet of the job]? In which case, I see it will not be for them, because they won’t get there… They’re their own worst enemy in that. There are obviously other reasons, and I can’t change home life or whatever, but that’s not going to go away as one moves through a training program. It doesn’t mean you’re bad, it doesn’t mean you’re incompetent, that’s a fact of life. [E9]

From the supervisor’s perspective, they regard themselves as trying their best to assist and, on this occasion at least, they appeared unaware of the “irritated” feelings roiling inside the trainee.

Supervisor annoyance did not always result in intensified circulating feelings. For example, a trainee stated (E22):

[This supervisor is] gruff and abrupt. You ring them on the phone and you think, “Holy shit, what did I bother ringing them for?” You get off the phone thinking, “Have I done something wrong?” They can have a short fuse …[but] once you can get past that outward façade… there is quite a caring, supportive individual under there

Here, despite the trainee’s immediate felt response to the supervisor’s tone, these feelings do not appear to intensify or stick or pull apart social relations between the supervisor and trainee. This aligns with the concept of the educational alliance (Telio et al., 2015), which suggests a known and trusted relationship may alter how feedback information is interpreted.

Information in feedback encounters is not only understood, it is *felt* through the prism of the day-to-day work. As the trainee in E9 notes:

I’m acutely aware that they’re disappointed in me as an individual …. because of the interactions I see with how the supervisor treats other people and then how the supervisor treats me …

The dynamic and constant intersection of feelings and judgements about performance is illustrated by peer interactions, which again are so informal but are laden with judgements about appropriate performance. The field researcher’s audio diary describes a group of trainees together:

Anyway, so there was that real closeness and– what’s the word? camaraderie? … and the conversation [between trainees] goes in circles and shifts, back and forward. Then peers are talking about another trainee, remarking that they shouldn’t have taken the day off…Then the participant said, “*Well*,* I took a day off yesterday.*” The peers respond [by reassuring the participant that it was ok to take a day off if you had a sick child].

This encounter reveals the constant moment by moment dynamics of social relations –a series of constant adjustments to feelings and judgements even within a warm, comradely conversation. It underlines the precarity of training and how the desire to succeed imbues even the most informal of feedback encounters.

Taken together, this exploration of our data suggests how *strangely* feelings permeate judgements. By strange, we mean that how and what people feel is unpredictable and indeterminate because, as Massumi (2021) suggests, feelings are beyond an individual and characterised by their openness to change. Participants might not know how they are feeling. On one day, a person might respond to irritation with irritation and on another, with amusement. The relationship between thinking and feeling is not necessarily causal or consistent or conscious (Massumi, 2021). However, we can make some overarching comments beyond this observation of unpredictability. In day-to-day feedback encounters, judgements about performance and feelings were co-produced and inseparable. They were premised on layers of social interactions that occur in the day-to-day work of clinical practice. Trainees particularly masked their feelings (and judgements). We noted that feelings tended to be exacerbated by the social hierarchies and that attempts at hiding feelings may have heightened asymmetries in power. Thus, the formal assessment and feedback moments, where both parties were most acutely aware of the power differentials, could strongly intensify feelings in some circumstances.

## Feelings and formal feedback encounters

Our fieldwork included several formal feedback encounters within mid-term and end-of-term assessments. These came with a different tenor to the day-to-day judgements experienced in daily clinical work. Participants appeared more dismissive of positive statements within these formal feedback encounters, in contrast to the moments of reassurance about day-to-day work that appeared to stick with trainees. For example, in E23, the trainee was told in a formal session:

We’re happy with your progress. We can’t think why you’re not sitting the exam yet. We think that you are at a stage where you should be sitting.

But the trainee ignored this advice, preferring their own view that they were not ready. In a similar vein, in E22, a trainee who had been previously critiqued, was praised for recent work:

From my point of view, it was nice to hear but in terms of, “Do I think I made massive changes to the way I go about my daily work.” My answer would be no. I feel like [I’m] still the same person but maybe it was just noticed a bit more. (E22)

In both of these reports, the supervisor commentaries did not alter the feelings of the trainee – they did not seem to stick or circulate. In E22, in particular, we see how the praise does not appear to have ameliorated the sense of trainee resentment. This casts a new light on Watling and colleague’s (2012) work on how learners weigh up the value of feedback information dependent on the source. They write: “credibility judgements about feedback are also influenced by the learner’s respect for the source of the feedback…” It helps us appreciate that credibility judgements, like other judgements about performance, are co-constructed in complex, contradictory and dynamic ways with feelings.

If a trainee was already aware of what the supervisor was going to say, then this also helped to reduce the intensity of feelings. For example, one trainee compared a heavily critical feedback session with another. They noted:

The big difference, I guess, between this feedback session and last feedback session is the stuff for me to improve on or to continue to work on or do, is not brought up brand new for the six-month mark about any of my failings, it was nothing new. (E22)

This contrasted with other formal critiques, where the shock of an unknown criticism or “blindsiding” (Bearman et al., 2023), intensified feelings. For example, in E10, a trainee reported being “told off” noting that it was “just out of blue” and “it was quite upsetting”. These feelings rapidly intensified, leading to despair. For example, a trainee said:

…it left me all very lost and contemplating whether or not this is what I wanted to keep doing. I don’t know how many more of these feedback sessions - [how much more] complete feeling like a failure- I will have in me before I completely walk away… (E0)

The intensity of these feelings galvanised the trainee into action. We saw with these heavily critical and surprising feedback sessions, trainees sought either alternative perspectives, support from senior colleagues, changes to training locations or seriously discussed resigning from the training program with their partner. Moreover, the force of the feelings that emerged within these formal feedback encounters did not go away. Fieldwork notes from E10 state: “The emotions … stayed with participants for months (maybe it is still with them).” This underlines how strongly these intensifying moments ‘stick’. They certainly stuck with us, the research team, amplifying our own discomfort during observation and analysis.

## Judgements about feelings

Another level of complication, and indeed strangeness, regarding feelings in feedback encounters, was that supervisors frequently made judgements about how trainees were or should be feeling. For example, trainees were told they were too “aggressive” (E1) or conversely “disinterested” (E10), or directed to be more “confident” (E18). These labels could circulate feelings within the trainee, often framed by trainees as confusion or disbelief or misgivings. In short, being directed to express their feelings differently prompted trainees to feel unsettled. Equally, they could be reassured when a supervisor told them that their feelings were legitimate. For example, a trainee expressed relief when told (E17): “your anxiety is appropriate. These are the decisions you will be paid for as a consultant.”

The gendered nature of some feedback encounters reinforced the socially prescribed nature of feelings in disturbing ways. Several women reported comments linking their “hormones” to displaying implicitly ‘wrong’ feelings – for example “coming as an angry mum …” or “caused my personality changes.” While the trainees seemed to shrug these off, if a little angrily, we as researchers were annoyed and distressed in reading them. By comparison, our analysis suggested that it was socially acceptable for trainees to be jokingly aggressive about anti-vaxxers (E3) or for a male supervisor to grumpily remark: “I couldn’t give a damn about that” (E9) to a trainee. von Shreve (2018) suggests that specific bodies, positional relations and dynamic feelings co-constitute each other, and these feedback encounters therefore exist within a “web” made of both gendered assumptions and individual actors. It also underlines the challenge presented by seeking to improve feedback in clinical environments.

We too became caught in the sticky web of judging/feeling/relating. An audio diary during data collection notes:

I don’t know if it was transference [of] what the participants was feeling I picked up on. I don’t know if it’s just the day that we’ve both had, and we’re both feeling that way… I felt like, “Oh my goodness. You’ve just had this -- you’re working so hard and then to be told, “You’re not doing good enough.“” … On the drive home, I was just thinking, “It just feels a bit–” hopeless isn’t the right word … just like, “Oh, for goodness sake,” yes. I really, really *felt* [sentence unfinished].

## If judgement itself is a matter of feeling, what does this mean for feedback?

In 2012, Eva and colleagues (p. 15) suggest: “The interplay observed between fear, confidence, and reasoning processes reinforces the notion that there is no simple recipe for the delivery of effective feedback.” A decade later, Ajjawi and colleagues (2022) comment in their literature review on emotions in feedback: “Emotions are not noise in the background to be ignored as conceptualised in the majority of papers; they are not merely internal but between and around us.” This study underlines both these contributions and extends them. Our key contention is that judgements about performance *must* come with feelings and even the very expression of neutrality (“non-judgemental”) is not devoid of feelings. We infer that the judgements made in and around feedback – such as credibility judgements, judgements about performance, judgements about how much to divulge and judgements about future actions – are themselves fundamentally entangled with feelings.

If judgement-making entails feelings (and vice-versa), what does this mean about our attempts to make and express judgements of performance? If a heavy sigh is a tacit judgment, how can we account for it? If a judgement is laden with the embodied, relational and social, can it be trusted? We propose that acknowledging and reflecting on the inevitable feelings that surround a judgement about performance (what Ajjawi and colleagues. (2022) call *emotional reflexivity*) is more productive than ignoring and potentially intensifying them. So, we suggest that talking about feelings should be somehow legitimised in feedback encounters and even in surrounding discussions about judgements like competency committees. But at the same time, we recognise the challenge for emotional reflexivity: a time-poor, high-stakes working environment, where feelings are both omnipresent and minimised.

Our observations of feedback encounters took place in often heavily charged settings, with time-pressures, emotional families or worry about clinical decisions. We noted: long working hours; unexpected patient death or harm to patients; the pressures of looking for a permanent position; and a range of personal circumstances that weighed heavily upon participants. We observed both strong camaraderie and feelings of isolation. We heard trainees accounts of *the worry* implicit in trying to jump over the hurdles that are part of the fabric of formal training programs. Even if they made the grade, were they suited to a career with this degree of ongoing uncertainty? Trainees reported tenuous links between their own practice and the outcomes of patients, and at the other extreme, knowledge that in certain specialities their own decision-making and actions could have dire consequences. Grief and loss were present in our trainees’ lives, as well as in the lives of their patients. We contend that the feelings of being in a healthcare environment tasked with heavy responsibilities - became entangled with the feelings in feedback encounters. But we did not observe this entanglement being explicitly addressed within feedback encounters except as normative judgement as to what the trainee should be feeling. Other feelings remained firmly unspoken in feedback dialogues. We read this as aligning with a discourse of ‘emotions to be managed’. And this is a pervasive mode.

Despite this, feelings were tacitly acknowledged in embodied and relational ways. Responses appeared *felt* rather than articulated, for example, warmth for the ICU trainee whose patient had died unexpectedly and tacit support for personally difficult circumstances. A notable exception highlights the possibility of the alternative. Our fieldwork included a highly positive extended pastoral dialogue, immediately *after* a formal feedback encounter, facilitated by a senior clinician who was “super supportive” and “family focussed”. This longer discussion allowed the trainee to express their feelings, particularly their personal situation and the impact it was having on their training:

I said, *I’m just finding it too much.* I just said, *look*,* I’m exhausted*. (E0)

The trainee raised this as a conversation that was exceptional, following a recommendation of a peer to have a conversation with a particular senior clinician. Moreover, it was associated with a label of underperformance attached to the trainee.

This leads us to reflect on how accepted it is that feelings should be avoided in feedback conversations, despite – or possibly because of – the inherent emotionality of clinical practice that permeates clinical environments. There is an irony that much of the contemporary feedback literature describes the primacy of learners tuning into cues (or information or ‘things’) within them and around them to help them navigate the world of work (Bearman et al., 2023; Van Der Leeuw et al., 2018; Watling et al., 2012). But how much should trainees and supervisors engage with this embodied unpredictability associated with reading the “atmosphere”? We are not certain: while navigating interpersonal dynamics and ‘reading the room’ is a big part of working and learning, attuning too much to how others are feeling might make trainees and supervisors hypervigilant.

Simply raising feelings in clinical environments may be a crucial first step towards feedback practices that take account of feelings. The immediate need is to provide forums to legitimate expression of emotions. We see this in other arenas, for example learners immediately talk about their feelings post a stressful simulation in a ‘reactions’ phase (Rudolph et al., 2008). In Dutch general practice training, van Braak and colleagues (2023) describe how trainees discuss together “what is affecting them?” at present. The results of this study suggest that the intense, emotional clinical situations that might be expected to prompt expressions of feelings, are not necessarily the ones that do. This underscores our point about the strangeness of feelings and provides ballast to our suggestion that people cannot be too interventionalist about their feelings. In other words, we don’t know what is going to affect us and when it does, it may not be clear how we work with our feelings to help us make sense of our interactions with others.

We like van Braak and colleagues’ very simple model of discussing feelings because it legitimises emotions as central but also avoids the label of the ‘pastoral conversation’. Of course, pastoral conversations are very important; the one reported in our data felt like it was the first moment the trainee was heard. However, our concern is that the label of pastoral may ‘hide’ feelings by labelling them as a personal matter. And this could become ‘stigmatising’ precisely because such feelings are hidden (Castanelli et al., 2024). Indeed, we suggest given the type of gendered comments we noted, future work might consider how gender imbues what feelings are permissible for whom, and which – for example, tears for a man or aggression for a woman - are stigmatised within feedback conversations.

So, given all this complexity and a social setting that minimises emotions, what are the practical implications about feedback in clinical environments? As this study reveals, feelings in feedback are very peculiar. They can disappear or sustain or amplify, and they mobilise things in them and around them. And they interact with their contexts: they are legitimised and delegitimised by social structures. We contend that while this means that a learner cannot put their feelings away, it also means that feelings are not their sole responsibility. Perhaps one way forward is to shift the locus of concern about feelings *beyond* the trainee. Clearly, supervisors also have emotional responses, and the irritation felt by our supervisors was palpable. The simulation literature proposes, in order for a teacher to make a ‘good judgement’, that they must hold the learners in high regard (Cheng et al., 2016). But part of the problem here is, as we see in this data, an articulated regard may not be a *felt* one. And while supervisors are much less vulnerable, given their status and security of their position, they might not necessarily feel that they are. As we keep repeating – echoing others (Aryal et al., 2015; Massumi, 2021) – feelings are strange, unpredictable and only sometimes conscious. Masking feelings may come at a cost for supervisors and trainees alike. Perhaps an even better target would be extending the responsibilities for feeling more broadly across the actors in the environment. This may be one of the advantages of coaches (Armson et al., 2019) or external observers (Fraser, 2007) – the different roles permit different types of feelings. But we wonder what would happen if the target was not just the trainee, but rather offering opportunities to acknowledge and discuss feelings with trainees, colleagues and supervisors alike?

### Strengths and limitations

We end with our own feelings about this research. It was difficult, challenging and evoked, as mentioned, many feelings including sadness and anger, but also pleasure in the meaning we were making. We are alive to the depth of the data we collected, and the value of observational and interview data, but also to the limitations of our approach. Longer time in many more clinical environments with a broader number of participants would have allowed us to tell some stories more fully. We worked hard to avoid inadvertently identifying our participants, and in so doing, may have sanitised and decontextualised their stories. We also recognise that much data collection took place during the pandemic. Although cases in Australia were limited, the possibility of uncontrolled outbreaks and socially isolating preventative restrictions made it an emotionally difficult time for many. However, in their feedback encounters and across our fieldwork, participants reported relatively limited distress directly related to the pandemic. There was, however, acknowledgement of the general uncertainty, particularly around their training programs, and deep difficulty of separation from loved ones due to travel bans.

## Conclusions

This post-qualitative ethnographic study sought to understand the interplay of emotions and feedback. Drawing on Massumi (2021) and Ahmed (2013) to recognise emotions as complex, social and relational, we identified how judgements are saturated with feelings which circulate and, on many occasions, amplify or stick. The work of emotions in judgements can be to reinforce social hierarchies, strengthen supervisory relationships, and manifest action even while supervisors assumed a neutral tone. Judgements *about* emotion could be normative and gendered. While the intensification of feelings during formal feedback is unavoidable, in the day to day we might start to raise feelings and pay attention to the work they are doing. This airing might avoid further intensification and potentially build better bonds between trainees and supervisors through their shared humanity.

## Data Availability

This data has the potential to identify participants and is therefore not provided.

## References

[CR1] Ahmed, S. (2013). *The cultural politics of emotion*. Routledge.

[CR2] Ajjawi, R., Olson, R. E., & McNaughton, N. (2022). Emotion as reflexive practice: A new discourse for feedback practice and research. *Medical Education*, *56*(5), 480–488. 10.1111/medu.14700 34806217 10.1111/medu.14700 PMC9299671

[CR3] Andreassen, P., Christensen, M. K., & Møller, J. E. (2020). Focused ethnography as an approach in medical education research. *Medical Education*, *54*(4), 296–302. 10.1111/medu.14045 31850537 10.1111/medu.14045

[CR4] Armson, H., Lockyer, J. M., Zetkulic, M., Könings, K. D., & Sargeant, J. (2019). Identifying coaching skills to improve feedback use in postgraduate medical education. *Medical Education*, *53*(5), 477–493. 10.1111/medu.13818 30779210 10.1111/medu.13818

[CR5] Aryal, Y., Boehler, A., & Brunner, C. (2015). Preface. In B. Massumi (Ed.), *Politics of affect*. John Wiley & Sons.

[CR8] Bearman, M., Dracup, M., Garth, B., Johnson, C., & Wearne, E. (2022). Learning to recognise what good practice looks like: How general practice trainees develop evaluative judgement. *Advances in Health Sciences Education*, *27*(1), 215–228. 10.1007/s10459-021-10086-3 34859317 10.1007/s10459-021-10086-3

[CR6] Bearman, M., Ajjawi, R., Castanelli, D., Denniston, C., Molloy, E., Ward, N., & Watling, C. (2023). Meaning making about performance: A comparison of two specialty feedback cultures. *Medical Education, 57*(11), 1010–1019. 10.1111/medu.15118 10.1111/medu.15118 37142553

[CR7] Bearman, M., Dracup, M., Ajjawi, R., Kirby, C., & Brown, J. (2024). Feedback, learning and becoming: Narratives of feedback in complex performance challenges. *Medical Education, 59*(2), 164–172. 10.1111/medu.15469 10.1111/medu.15469 PMC11708812 38978135

[CR9] Bing-You, R., Varaklis, K., Hayes, V., Trowbridge, R., Kemp, H., & McKelvy, D. (2018). The feedback tango: An integrative review And Analysis of the content of the teacher–learner feedback exchange. *Academic Medicine*, *93*(4), 657–663. 10.1097/ACM.0000000000001927 28991848 10.1097/ACM.0000000000001927

[CR10] Brown, S. D., & Stenner, P. (2001). Being affected: Spinoza and the psychology of emotion. *International Journal of Group Tensions*, *30*, 81–105.

[CR11] Bynum, W. E. I., Artino, A. R. J., Uijtdehaage, S., Webb, A. M. B., & Varpio, L. (2019). Sentinel emotional events: The Nature, Triggers, and effects of shame experiences in medical residents. *Academic Medicine*, *94*(1), 85–93. 10.1097/acm.0000000000002479 30277959 10.1097/ACM.0000000000002479

[CR12] Cantillon, P., De Grave, W., & Dornan, T. (2022). The social construction of teacher and learner identities in medicine and surgery. *Medical Education*, *56*(6), 614–624. 10.1111/medu.14727 34993973 10.1111/medu.14727 PMC9305233

[CR13] Carless, D., & Boud, D. (2018). The development of student feedback literacy: Enabling uptake of feedback. *Assessment & Evaluation in Higher Education*, *43*(8), 1315–1325. 10.1080/02602938.2018.1463354

[CR15] Castanelli, D. J., Weller, J. M., Molloy, E., & Bearman, M. (2022). Trust, power and learning in workplace-based assessment: The trainee perspective. *Medical Education*, *56*(3), 280–291. 10.1111/medu.14631 34433230 10.1111/medu.14631 PMC9292503

[CR14] Castanelli, D. J., Molloy, E., & Bearman, M. (2024). The stigma of underperformance in assessment and remediation. *Advances in Health Sciences Education, 30*, 815–830. 10.1007/s10459-024-10382-8 10.1007/s10459-024-10382-8 PMC12119712 39412554

[CR16] Cheng, A., Morse, K. J., Rudolph, J., Arab, A. A., Runnacles, J., & Eppich, W. (2016). Learner-Centered debriefing for health care simulation education: Lessons for faculty development. *Simulation in Healthcare*, *11*(1), 32–40. 10.1097/sih.0000000000000136 26836466 10.1097/SIH.0000000000000136

[CR17] Cheshire, L. (2016, July 17–20). *Thinking big (and critically) about qualitative research: Trends, visions and challenges for the future of qualitative social science*. Keynote Address at the Australian Consortium of Social and Political Research (ACSPRI) Annual Conference, The University of Sydney, Sydney, Australia.

[CR18] Coelho, V., Scott, A., Bilgic, E., Keuhl, A., & Sibbald, M. (2022). Understanding feedback for learners in interprofessional settings: A scoping review. *International Journal of Environmental Research and Public Health*, *19*(17), 10732. https://www.mdpi.com/1660-4601/19/17/10732 36078451 10.3390/ijerph191710732 PMC9517787

[CR19] Dennis, A. A., Foy, M. J., Monrouxe, L. V., & Rees, C. E. (2018). Exploring trainer and trainee emotional talk in narratives about workplace-based feedback processes. *Advances in Health Sciences Education*, *23*(1), 75–93. 10.1007/s10459-017-9775-0 28456856 10.1007/s10459-017-9775-0 PMC5801389

[CR20] Esterhazy, R. (2018). What matters for productive feedback? Disciplinary practices and their relational dynamics. *Assessment & Evaluation in Higher Education*, *43*(8), 1302–1314. 10.1080/02602938.2018.1463353

[CR21] Eva, K. W., Armson, H., Holmboe, E., Lockyer, J., Loney, E., Mann, K., & Sargeant, J. (2012). Factors influencing responsiveness to feedback: On the interplay between fear, confidence, and reasoning processes. *Advances in Health Sciences Education*, *17*(1), 15–26. 10.1007/s10459-011-9290-7 21468778 10.1007/s10459-011-9290-7 PMC3274671

[CR22] Fox, N. J. (2015). Emotions, affects and the production of social life. *The British Journal of Sociology*, *66*(2), 301–318. 10.1111/1468-4446.12119 25788237 10.1111/1468-4446.12119

[CR23] Fraser, J. (2007). Registrar clinical teaching visits: Evaluation of an assessment tool [Journal Article]. *Australian Family Physician*, *36*(12), 1070. 10.3316/informit.355803253979484. https://search.informit.org/doi/pdf/18075639

[CR24] Gingerich, A., Sebok-Syer, S. S., Lingard, L., & Watling, C. J. (2022). The shift from disbelieving underperformance to recognising failure: A tipping point model. *Medical Education*, *56*(4), 395–406. 10.1111/medu.14681 34668213 10.1111/medu.14681

[CR25] Hoeyer, K., & Bearman, M. (2023). The why, the what and the so what of a qualitative empirical research Article [Journal Article]. *Focus on Health Professional Education: A Multi-Professional Journal*, *24*(3), 95–105. 10.3316/informit.374250506938389. https://search.informit.org/doi/pdf/

[CR26] Jackson, A. Y., & Mazzei, L. (2011). *Thinking with theory in qualitative research: Viewing data across multiple perspectives*. Routledge.

[CR27] Jensen, L. X., Bearman, M., & Boud, D. (2023). Feedback encounters: Towards a framework for analysing and Understanding feedback processes. *Assessment & Evaluation in Higher Education*, *48*(1), 121–134. 10.1080/02602938.2022.2059446

[CR28] Knoblauch, H. (2005). Focused ethnography. *Forum Qualitative Sozialforschung / Forum: Qualitative Social Research*, *6*(3). 10.17169/fqs-6.3.20

[CR29] Lather, P., & St. Pierre, E. A. (2013). Post-qualitative research. *International Journal of Qualitative Studies in Education*, *26*(6), 629–633. 10.1080/09518398.2013.788752

[CR30] Lipnevich, A. A., & Panadero, E. (2021). A review of feedback models and theories: Descriptions, definitions, and conclusions [Review]. *Frontiers in Education*, *6*. 10.3389/feduc.2021.720195.

[CR31] Massumi, B. (2021). *Parables for the virtual: Movement, affect, sensation*. Duke University Press.

[CR32] Mehrabi, T. (2020). Almanac: Affective method. *Matter: Journal of New Materialist Research*, *2*, 154–157. 10.1344/jnmr.v1i2.31973

[CR33] Olson, R. E., Bellocchi, A., & Dadich, A. (2020). A post-paradigmatic approach to analysing emotions in social life. *Emotions and Society*, *2*(2), 157–178. 10.1332/263169020X15893854268688

[CR34] Ramani, S., Könings, K. D., Ginsburg, S., & Van Der Vleuten, C. P. (2020). Relationships as the backbone of feedback: Exploring preceptor and resident perceptions of their behaviors during feedback conversations. *Academic Medicine*, *95*(7), 1073–1081. 10.1097/ACM.0000000000002971 31464736 10.1097/ACM.0000000000002971

[CR35] Rudolph, J. W., Simon, R., Raemer, D. B., & Eppich, W. J. (2008). Debriefing as formative assessment: Closing performance gaps in medical education. *Academic Emergency Medicine*, *15*(11), 1010–1016. 10.1111/j.1553-2712.2008.00248.x 18945231 10.1111/j.1553-2712.2008.00248.x

[CR36] Sargeant, J., Mann, K., Sinclair, D., Van der Vleuten, C., & Metsemakers, J. (2008). Understanding the influence of emotions and reflection upon multi-source feedback acceptance and use. *Advances in Health Sciences Education*, *13*(3), 275–288. 10.1007/s10459-006-9039-x 17091339 10.1007/s10459-006-9039-x

[CR37] St Pierre, E. A. (2014). A brief and personal history of post qualitative research: Toward “post inquiry”. *Journal of Curriculum Theorizing*, *30*(2).

[CR38] St. Pierre, E. A. (2019). Post qualitative inquiry in an ontology of immanence. *Qualitative Inquiry*, *25*(1), 3–16. 10.1177/1077800418772634

[CR39] Telio, S., Ajjawi, R., & Regehr, G. (2015). The educational alliance as a framework for reconceptualizing feedback in medical education. *Academic Medicine*, *90*(5), 609–614. 10.1097/ACM.0000000000000560 25406607 10.1097/ACM.0000000000000560

[CR40] van Braak, M., Schaepkens, S. P. C., van Dolder, E., Dral, L. K., van der Horst, Z., Houben, D. B., & Mees, E. E. (2023). What affects you? A conversation analysis of exploring emotions during reflection sessions in Dutch general practitioner training [Original research]. *Frontiers in Psychology*, *14*. 10.3389/fpsyg.2023.1198208 10.3389/fpsyg.2023.1198208 PMC10476000 37671103

[CR41] Van Der Leeuw, R. M., Teunissen, P. W., & Van Der Vleuten, C. P. (2018). Broadening the scope of feedback to promote its relevance to workplace learning. *Academic Medicine*, *93*(4), 556–559. 10.1097/acm.0000000000001962 29068817 10.1097/ACM.0000000000001962

[CR42] von Scheve, C. (2018). A social relational account of affect. *European Journal of Social Theory*, *21*(1), 39–59. 10.1177/1368431017690007

[CR43] Watling, C., Driessen, E., van der Vleuten, C. P. M., & Lingard, L. (2012). Learning from clinical work: The roles of learning cues and credibility judgements. *Medical Education*, *46*(2), 192–200. 10.1111/j.1365-2923.2011.04126.x 22239333 10.1111/j.1365-2923.2011.04126.x

[CR44] Weallans, J., Roberts, C., Hamilton, S., & Parker, S. (2021). Guidance for providing effective feedback in clinical supervision in postgraduate medical education: A systematic review. *Postgraduate Medical Journal*, *98*(1156), 138–149. 10.1136/postgradmedj-2020-139566 33563716 10.1136/postgradmedj-2020-139566

